# A multiscale spatial modeling framework for the germinal center response

**DOI:** 10.3389/fimmu.2024.1377303

**Published:** 2024-05-30

**Authors:** Derek P. Mu, Christopher D. Scharer, Norbert E. Kaminski, Qiang Zhang

**Affiliations:** ^1^ Montgomery Blair High School, Silver Spring, MD, United States; ^2^ Department of Microbiology and Immunology, School of Medicine, Emory University, Atlanta, GA, United States; ^3^ Department of Pharmacology & Toxicology, Institute for Integrative Toxicology, Center for Research on Ingredient Safety, Michigan State University, East Lansing, MI, United States; ^4^ Gangarosa Department of Environmental Health, Rollins School of Public Health, Emory University, Atlanta, GA, United States

**Keywords:** B cells, germinal center, dark zone, light zone, affinity maturation, proliferative burst, chemotaxis

## Abstract

The germinal center response or reaction (GCR) is a hallmark event of adaptive humoral immunity. Unfolding in the B cell follicles of the secondary lymphoid organs, a GC culminates in the production of high-affinity antibody-secreting plasma cells along with memory B cells. By interacting with follicular dendritic cells (FDC) and T follicular helper (Tfh) cells, GC B cells exhibit complex spatiotemporal dynamics. Driving the B cell dynamics are the intracellular signal transduction and gene regulatory network that responds to cell surface signaling molecules, cytokines, and chemokines. As our knowledge of the GC continues to expand in depth and in scope, mathematical modeling has become an important tool to help disentangle the intricacy of the GCR and inform novel mechanistic and clinical insights. While the GC has been modeled at different granularities, a multiscale spatial simulation framework – integrating molecular, cellular, and tissue-level responses – is still rare. Here, we report our recent progress toward this end with a hybrid stochastic GC framework developed on the Cellular Potts Model-based CompuCell3D platform. Tellurium is used to simulate the B cell intracellular molecular network comprising NF-κB, FOXO1, MYC, AP4, CXCR4, and BLIMP1 that responds to B cell receptor (BCR) and CD40-mediated signaling. The molecular outputs of the network drive the spatiotemporal behaviors of B cells, including cyclic migration between the dark zone (DZ) and light zone (LZ) via chemotaxis; clonal proliferative bursts, somatic hypermutation, and DNA damage-induced apoptosis in the DZ; and positive selection, apoptosis via a death timer, and emergence of plasma cells in the LZ. Our simulations are able to recapitulate key molecular, cellular, and morphological GC events, including B cell population growth, affinity maturation, and clonal dominance. This novel modeling framework provides an open-source, customizable, and multiscale virtual GC simulation platform that enables qualitative and quantitative *in silico* investigations of a range of mechanistic and applied research questions on the adaptive humoral immune response in the future.

## 1 Introduction

The adaptive humoral immune response is a vital component of host defense, where B cells terminally differentiate into plasma cells (PCs) that secrete antibodies specifically recognizing and neutralizing the invading foreign antigens. The B cell responses can be broadly classified into two types: T cell-dependent and independent, depending on whether helper T (Th) cells are involved (1). In the T cell-independent response, naive B cells are activated directly via toll-like receptors (TLR) recognizing pathogen components such as lipopolysaccharide (LPS) or CpG DNA or via B cell receptors (BCR), without the assistance of Th cells (2, 3). The response is launched quickly and can occur within a few days of initial infection. Upon activation, B cells undergo clonal proliferation and differentiate into PCs, which secrete pentameric IgM molecules. While these antibodies provide initial protection, they are often polyclonal, not highly specific, and the IgM-secreting PCs are short-lived, thus unable to provide long-term immunity. In contrast, the T cell-dependent B cell response takes longer to develop, but through affinity maturation and class switch recombination (CSR) it can produce long-lived PCs that can provide life-long immunity with high-affinity IgG or other non-IgM antibody classes (4). Additionally, memory B cells are generated during the primary response, which can quickly launch a secondary antibody response upon subsequent exposure to the same antigens (5).

T cell-dependent B cell activation can take place either extrafollicularly, e.g., at the T-B border (6), or intrafollicularly in the germinal centers (GC), which are specialized, often transient, microstructures formed in the B cell follicles of secondary lymphoid tissues such as lymph nodes and spleen in response to infection or immunization (7–10). GC B cells exhibit unique spatiotemporal dynamics (11, 12). A GC is polarized, containing two distinct, physically separated zones: the dark zone (DZ) and the light zone (LZ). In the DZ, B cells undergo clonal proliferative bursts, during which somatic hypermutation (SHM) occurs. During SHM, the hypervariable regions of the genes encoding the immunoglobulin heavy chains and light chains are point-mutated by activation-induced cytidine deaminase (AID) at a high rate (13). As a result, the BCR affinities of the participating B cell clones for the invading antigen are modified and diversified. During SHM those B cells incurring damaging mutations that prevent normal assembly of surface BCRs are killed via apoptosis in the DZ (14, 15). After exiting the cell cycle following a proliferative burst, B cells migrate from the DZ to LZ under the chemoattractant force by CXCL13 secreted by follicular dendritic cells (FDCs) in the LZ (16, 17).

In the LZ, two main cell types participate in the positive selection of B cell clones harboring immunoglobulin (Ig) gene variants encoding relatively high-affinity antibodies: the residential FDCs and CD4^+^ T follicular helper (Tfh) cells. These two cell types coordinate to provide key molecular signals for B cell activation, survival, DZ re-entry, proliferation, and differentiation (18–20). FDCs are antigen-presenting cells (APCs), which previously encountered and engulfed pathogens and present the antigen epitopes on their cell surface. When B cells first encounter FDCs, their BCRs are activated by the surface antigens of FDCs. B cells then internalize the antigen-BCR complex and present the antigen epitopes on their own surface through major histocompatibility complex (MHC) II molecules to form peptide-MHCII complex (pMHCII). The density of pMHCII on the cell surface is proportional to the BCR affinity. Stronger BCR signaling also leads to higher PI3K-AKT-FOXO1 signal transduction (18, 21, 22). When the B cells subsequently encounter Tfh cells, a complex mutual interaction occurs between the two cell types (19, 23, 24). Tfh cells are activated via T cell receptors (TCR) liganded by pMHCII of the B cells, as well as by other cell surface signaling molecules such as inducible co-stimulator ligand (ICOSL) (25). Activated Tfh cells in turn express surface CD40L which reciprocally activates B cells together with several secretory cytokines including interleukins (IL) 4, 10, and 21 (26–28). CD40 signaling leads to NF-κB activation, increasing the chance of survival of B cells. In the presence of downregulated FOXO1, NF-κB elicits transient MYC activation that initiates the cell cycle (18). Only a small fraction of B cells is positively selected, which express CXCR4, the receptor for chemokine CXCL12, and migrate back to DZ where they undergo further proliferative bursts and SHM (16, 22). Those B cells with weaker BCR affinity are more likely to undergo apoptosis in the LZ, as well as B cells that do not have a chance to encounter Tfh cells in the LZ. As a result of combined action of proliferation and SHM in the DZ and positive selection in the LZ, the overall BCR affinity of the GC B cell population for the antigen continues to improve. After many rounds of DZ-LZ cycles, a small fraction of B cells are affinity-matured and exit the GC as either long-lived antibody-secreting PCs or memory B cells.

The GC plays a critical role in the generation of long-term protective immunity, and this is relevant in both the context of natural infection and vaccination for infectious diseases such as COVID-19 (29). If the GC is compromised or cannot be sufficiently induced due to genetic alterations, increased susceptibility to bacterial and viral infections will result. On the other hand, unintentional recognition of self-antigens and induction of GC can lead to autoimmune diseases such as systemic lupus erythematosus (30). Dysregulated B cell proliferation in GC can lead to lymphoma or other B cells-related leukemia (31). The GC also plays a role in antibody-mediated rejection of transplanted organs (32). In addition, many environmental contaminants are immunotoxicants, some of which can suppress B cell activation and the humoral immune response, leading to increased susceptibility to infectious disease and cancer (33). Therefore, a full mechanistic understanding of the complexity of GC is crucially important for sustaining immune integrity and preventing or alleviating many pathological conditions.

Computational modeling has played a long-standing role in dissecting and understanding the complex dynamics of GC immune responses (34, 35). The GC involves an elaborate interplay between many cell types, signaling molecules, transcription factors, and actuator genes (36). Key signal transduction and gene regulatory networks underpin the spatiotemporal dynamics of GC B cells and are crucial for the positive selection and ultimate formation of high-affinity PCs. Although there have been many efforts simulating the cellular dynamics and affinity maturation of GCs, cross-scale modeling that integrates molecular, cellular, and tissue-level actions in a spatial context has only begun to emerge recently and thus is still rare (37–39). In this study, we presented a novel multiscale mathematical modeling framework of the GC developed in the CompuCell3D simulation environment that integrates the molecular network and spatiotemporal behaviors of GC B cells. The modeling framework provides an open-source, customizable, multiscale virtual GC platform that enables future *in silico* investigations of a range of questions both qualitatively and quantitatively, including B cell population turnover, BCR mutation rate, death timer, proliferative burst size, availability of Tfh cells, and effects of genetic and chemical perturbations.

## 2 Methods

### 2.1 Model structure

#### 2.1.1 Cell types, cellular events, and interactions modeled

Four major cell types involved in GCR are modeled in the framework: CXCL12-expressing reticular cells (CRCs) located in the DZ, FDCs and Tfh cells in the LZ, and B cells cycling between the DZ and LZ (7–10). For simplicity, CRC, FDC, and Tfh cells are treated as stationary. Key cellular events of B cells captured in the model include: (i) B cell volume growth, division, SHM, and apoptosis in the DZ; (ii) DZ-to-LZ B cell migration and simultaneous initiation of a cell death timer; (iii) interaction of B cells with FDCs in the LZ to determine BCR antigen affinity, interaction of B cells with Tfh cells in the LZ to make probabilistic decisions based on pMHCII density on positive selection, survival, initiation of cell growth, and DZ re-entry of positively selected B cells, and death timer-triggered apoptosis of LZ B cells not positively selected.

#### 2.1.2 Molecular events in B cells

**Figure 1:**
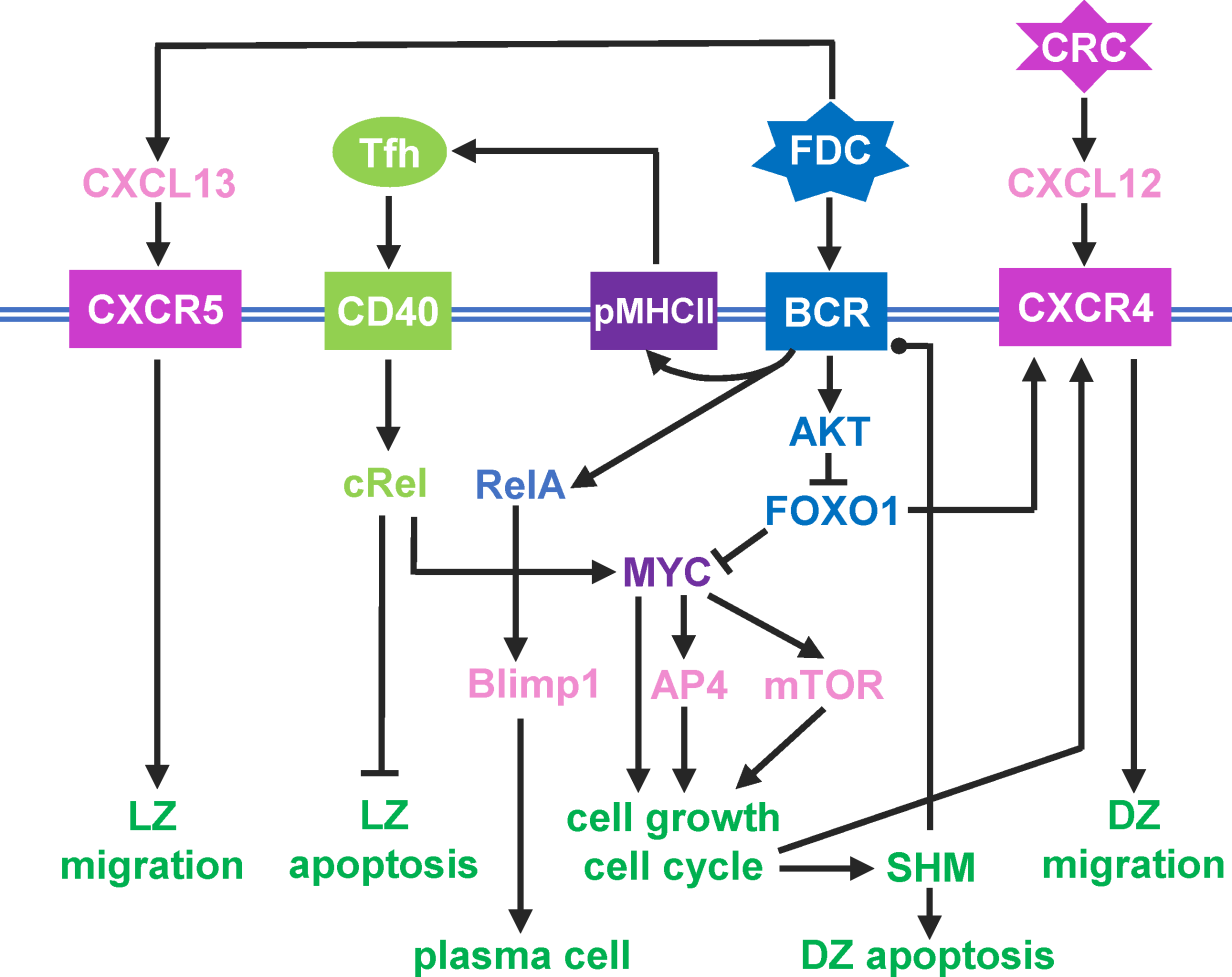
Schematic illustration of a simplified intracellular molecular network of GC B cells and different B cell outcomes driven by key molecules as indicated. Pointed arrowhead: stimulation/activation, blunted arrowhead: inhibition, and dotted arrow head: regulation in either direction.

The above cellular events are driven by an intracellular molecular network in B cells that responds to diffusive chemoattractants and signaling molecules from FDCs and Tfh cells. For simplicity, the following molecular species and regulatory events are included in the model (**Figure 1**). A DZ-to-LZ descending gradient of chemoattractant CXCL12 is established by CRCs in the DZ (40, 41), and an opposite gradient of chemoattractant CXCL13 is established by FDCs in the LZ (17, 42). In the LZ, the contact of a B cell with an FDC will trigger BCR-mediated signal transduction, which leads to several signaling events in the modeled B cell: (i) re-expression on B cell surface of pMHCII, the density of which depends on BCR affinity for the antigen, (ii) transient activation of AKT and downregulation of FOXO1, the extent of which depends on BCR affinity (18, 21, 22), (iii) once the BCR affinity reaches a threshold, a switch-like activation of NF-κB subtype RelA is triggered (43–45), which in turn induces BLIMP1 (46, 47), leading to terminal B cell differentiation into antibody-secreting PCs.

If a B cell expressing pMHCII encounters a Tfh cell, the B cell is stimulated via CD40 signaling which activates another NF-κB subtype, cRel. cRel activation leads to at least two molecular signaling events. It activates Bcl-xL which inhibits apoptosis, thus terminating the death timer (48). With FOXO1 still downregulated, cRel also induces the expression of MYC (18). Upregulation of MYC triggers a commitment to cell growth and initiates the cell cycle (49). MYC also activates AP4, which sustains B cell growth and division burst (50). With the B cell committed to growth and proliferation, as FOXO1 is re-expressed, CXCR4 is induced (22, 51). As a result, the B cell migrates towards the DZ in response to the CXCL12 gradient. In the DZ, shortly after each cell division, each of the two daughter cells incurs an independent point mutation of the BCR, which alters its affinity for the antigen with some probabilities (52–54). The probability of a damaging mutation is encoded such that a fraction of B cell progeny dies by apoptosis in the DZ (14, 15). The surviving B cells will continue to grow and divide as long as AP4 remains above a threshold (50). When AP4 drops below the threshold, the B cells exit the cell cycle, followed shortly by downregulation of CXCR4 (55). With CXCR5 constitutively expressed (12, 16), the B cells will be pulled by the CXCL13 gradient field into the LZ, repeating the DZ-LZ cycle.

#### 2.1.3 Model assumptions and simplification

The molecular and cellular events involved in the GC are complex. Here, for the purpose of modeling, several assumptions and simplifications are made.

- The mutual activation between B cells and Tfh cells is simplified to pMHCII density-dependent CD40-cRel activation, as described above.
- Many factors involved in GC, including BCL6, BACH2, and IRF4, are not explicitly considered.
- Initial migration of B cells from the T/B cell border towards the LZ is not considered and neither is the Tfh cell migration into the LZ. CSR is thus not included as it is believed to occur primarily during pre-GC formation (56).
- The initial clonal expansion of GC seeder B cells without SHM and positive selection is not considered for the lack of clear known mechanisms that terminate this initial proliferative burst phase and start the subsequent competitive selection phase.
- For B cells returning to the DZ, a delay variable is introduced before the cell growth for the first cell cycle is initiated to reduce the chance of cell division in the LZ.
- The GC exit of PCs is modelled as deleting these cells from the simulation once they emerge.
- Formation of memory B cells is not considered.

### 2.2 Construction of the computational model in CompuCell3D

The GC model was constructed and simulated as a hybrid, agent-based stochastic model in CompuCell3D. CompuCell3D provides a flexible and customizable platform for simulating multi-cellular behaviors and interactions based on the Glazier-Graner-Hogeweg approach (57). The Cellular Potts Model module in CompuCell3D was employed to simulate the physical properties and movements of individual B cells (58), while the molecular network operating in each individual B cell, as depicted in **Figure 1**, was simulated by using the Gillespie’s stochastic algorithm implemented in Tellurium conforming to the Antimony notation (59, 60). The CompuCell3D model consists of four files: an XML file, a Potts initialization file (PIF), and two Python script files. The XML file contains various “Plugins” and “Steppables” that define some default Potts model parameter values. The *Chemotaxis* plugin defines CXCL12 and CXCL13 as the chemoattractants, and the *DiffusionSolverFE* steppable designates that CXCL12 and CXCL13 are secreted by CRC and FDC, respectively and specifies the parameter values for secretion, diffusion, and decay in the Medium. The PIF file contains the initial coordinates of medium and cells where applicable. The steppable Python file contains the script that defines several steppable classes, including *GCR_Steppable*, *MitosisSteppable*, *BCell_GRNSteppable*, and *VisualizationSteppable*, and the Tellurium model. The model is initialized in the “start” section of *GCR_Steppable*, including the generation of CRCs, FDCs, Tfh cells, and seeding B cells. Each B cell is assigned a Tellurium molecular network model named as *BcellNetwork*.

#### 2.2.1 Cell-cell contact and probabilistic decision-making

To capture the physical contact between B cells, FDCs, and Tfh cells, the *NeighborTracker* and *PixelTracker* plugins are employed to identify the neighboring cells of each B cell. Once a contact with an FDC is identified, the pMHCII level of the B cell is set proportional to its antigen-specific BCR affinity. Upon subsequent contact with a Tfh cell, the B cell can be positively selected based on a probability that is proportional to the pMHCII level. For the positively selected cell, the running death timer is terminated, cell cycle is committed, and DZ re-entry is initiated.

#### 2.2.2 SHM and probability of BCR affinity alteration

Each of the two daughter cells of a dividing B cell has a probability of 0.3 to produce a damaging mutation that will result in cell death in the DZ. For the daughter cell that does not incur a damaging mutation, an SHM can either increase, decrease, or does not change the BCR affinity, each with a probability of 1/3. The increment or decrement of the affinity alteration can be either 0.25 or 0.5 with equal probability. In general, the BCR affinity ranges between 0–10, but can be higher.

### 2.3 Simulation data collection, storage, analysis, and model sharing

Variables of each B cell are saved in a plain text file which is updated every 15 Monte Carlo steps (*mcs*). The file is named in the format of “Generation_mother ID_cell ID.txt” to facilitate lineage tree construction. The CompuCell3D model files containing the parameter values and Python script for analysis of simulated data are available at https://github.com/pulsatility/2024-GCR-Model.git.

## 3 Results

### 3.1 Morphology of a simulated GC

**Figure 2:**
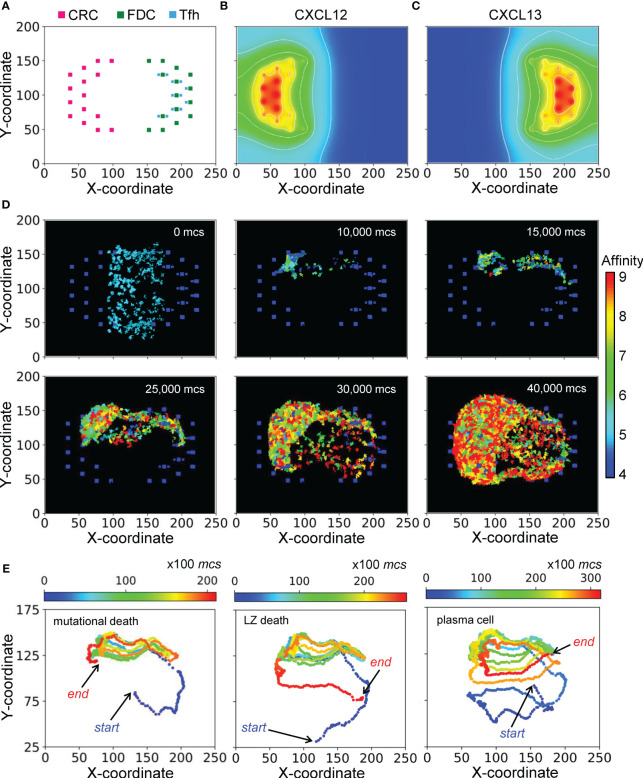
Morphology of a representative simulated GC. **(A)** 2-D distributions of CRCs, FDCs, and Tfh cells in the DZ (left) and LZ (right) with X-Y coordinates indicated. **(B, C)** Concentration gradients of chemoattractants CXCL12 and CXCL13 respectively. White contour lines: isolines of equal concentrations. **(D)** Snapshots of simulated GC at various *mcs* indicated. Colors of B cells denote BCR/antibody affinity as indicated by the colormap. **(E)** 2-D trajectories of B cells in three select lineages leading respectively to damaging mutation-induced apoptosis in DZ (left panel), death timer-triggered apoptosis in the LZ for not positively selected (mid panel), and emergence of a PC (right panel). Dot color denotes *mcs* time as indicated by the colormap.

The morphological results of a representative simulation of the GC model are shown in **Figure 2**. The simulated space dimension is 250x200x11 pixels, which can be considered as 250x200x11 in µm in real space, to represent a slice of the GC to save computational time. The 2-D projection of the instantiated non-B cells on the X-Y plane is indicated in **Figure 2A**, while along the Z dimension, these cells are distributed randomly in 3 of the 11 layers (results not shown). There are 45 CRCs distributed on the left half of the field, which becomes the future DZ, and 45 FDCs and 36 Tfh cells distributed on the right half of the field, which becomes the future LZ. Tfh cells are located next to FDCs, reflecting the notion that they also express CXCR5 and are thus drawn to FDCs which secrete chemoattractant CXCL13 (61, 62). Not all FDCs are surrounded by Tfh cells, mimicking the situation that the availability of Tfh cells is a limiting factor in the positive selection of LZ B cells (12, 63–65). CRCs and FDCs secrete CXCL12 and CXCL13 respectively, establishing two opposing chemoattractant fields and thus the polarity of the GC (**Figures 2B, C**).

With the layout of residential cells and chemoattractant fields as above, a simulated GC that succeeds in producing B cells of high BCR/antibody affinities is shown as snapshots in **Figure 2D** and in **Supplementary Video S1**. For this simulation, the GC starts with 200 B cells (clones) of intermediate affinity of 5 (indicated by the color of the cells) between the DZ and LZ. Over a period of 40,000 Monte Carlo steps (*mcs*, where 100 mcs can be regarded approximately as 1 hour in real time), both the number of total GC B cells and the fraction of high-affinity B cells increase, indicating successful GC population growth and affinity maturation. The 2-D trajectories of 3 select B cell lineage branches leading to different cell fates are shown in **Figure 2E**. These trajectories cycle between the DZ and LZ for multiple rounds. The first trajectory ends with cell death in the DZ due to damaging mutation during mitosis (left panel), the second trajectory also ends with cell death but in the LZ due to death timer (mid panel), and the last trajectory ends with differentiation into a PC in the LZ (right panel).

### 3.2 Cellular events of GC B cells

#### 3.2.1 B cell population dynamics

**Figure 3:**
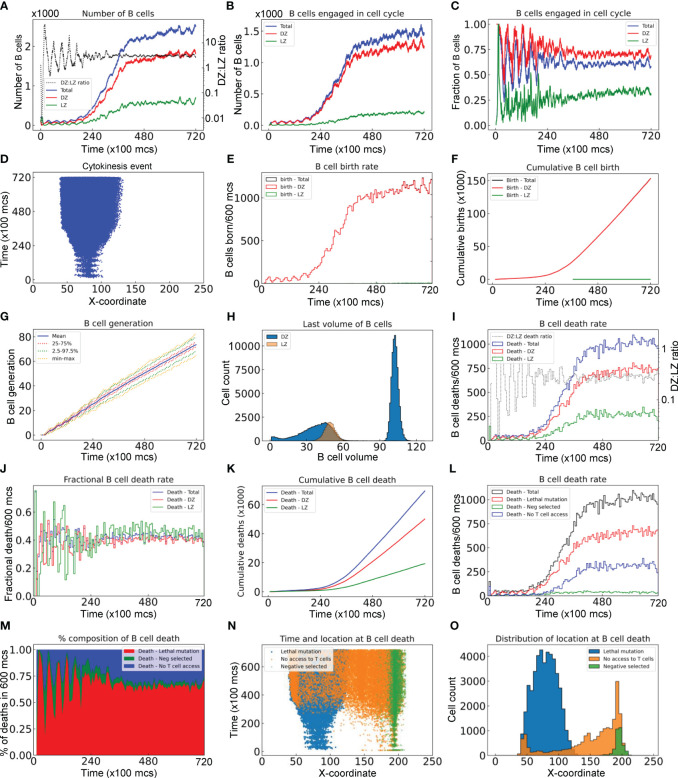
Quantitative analyses of B cell population dynamics in a simulated GC. **(A)** Numbers of B cells in the DZ, LZ, and total, and DZ: LZ ratio as indicated at a given time. **(B)** Numbers of B cells engaged in cell cycle in DZ, LZ, and total as indicated. **(C)** Fractions of B cells in the DZ, LZ, and total that are engaged in cell cycle. **(D)** The X-coordinate and time at which cytokinesis events occur. **(E)** Numbers of B cells born every 600 *mcs* in the DZ, LZ, and total as indicated. **(F)** Numbers of cumulative cell births in the DZ, LZ, and total as indicated. **(G)** Mean, interquartile, 2.5–97.5^th^ percentile, minimum and maximum generations of GC B cells as indicated. **(H)** Distributions of last volumes of B cells in the DZ and LZ as indicated before they divide, die, or differentiate into PCs. **(I)** Numbers of B cell deaths in every 600 *mcs* in the DZ, LZ, and total, and DZ: LZ death ratio as indicated. **(J)** Fractions of B cells in the DZ, LZ, and total that die in every 600 *mcs* as indicated. **(K)** Numbers of cumulative cell deaths in the DZ, LZ, and total as indicated. **(L)** Numbers of B cell deaths in every 600 *mcs* in total, due to damaging BCR (lethal) mutation, not being positively (Neg) selected after contacting Tfh cells, or no access to Tfh cells as indicated. **(M)** Percentage composition of B cell deaths in every 600 *mcs* due to lethal mutation, negative selection, or no access to Tfh cells as indicated. **(N)** The X-coordinate and time at which cell deaths occur. **(O)** Distributions of X-coordinate at which cell deaths occur due to lethal mutation, negative selection, or no access to Tfh cells as indicated.

In this section we performed an in-depth quantitative analysis of the GC B cell population dynamics with respect to time, location, and cell fates. For the GC simulation presented in **Figure 2**, the total number of B cells (*N_Tot_*) increases rapidly from the initial 200 over a period of 40,000 *mcs*, then approaches a steady-state size of about 2,500 cells through 72,000 *mcs* (equivalent to 30 days) without considering GC termination (**Figure 3A**). The growth of the B cell population is not smooth – it proceeds in an uneven fashion due to random births and deaths occurring simultaneously. In the early stage of the GC, the numbers of B cells in the DZ (*N_DZ_*) and LZ (*N_LZ_*) alternate in anti-phase, resulting from cyclic cell migration in unison between the two zones (Video S1). In the late stage, *N_DZ_* is persistently greater than *N_LZ_* with the *N_DZ_: N_LZ_* ratio stabilizing near 3:1 (**Figure 3A**). The evolving B cell population in the GC is highly dynamic with constant turnover through several processes. Specifically, (i) B cells are born in the DZ as a result of proliferative bursts of clonal expansion; (ii) B cells are cleared from the GC via apoptosis triggered by damaging BCR mutations in the DZ and via apoptosis in the LZ if not positively selected; and (iii) B cells exit the GC as PCs. We next quantified the birth and death (apoptosis) events.

##### 3.2.1.1 B cell birth

The number of B cells engaged in cell cycle increases over time and these cells are predominantly in the DZ (**Figure 3B**). There are a small number of cell cycle-engaged B cells in the LZ, representing positively selected B cells that just initiate the cell cycle without much growing yet. Approaching the steady state, nearly 70% and 30% of DZ and LZ B cells, respectively, are engaged in the cell cycle, while on average 65% of the overall B cell population is in the cell cycle (**Figure 3C**). B cells are born predominantly in the DZ with only a negligible number of births in the LZ (**Figure 3D**). The absolute birth rate increases over time approaching about 1100 births per 600 *mcs* (**Figure 3E**). Cumulative births reach 150K in the entire 72,000 *mcs* period when two birth events are registered for each cell division (**Figure 3F**). The mean cell generation increases almost linearly with time while the variability also progressively increases as more B cells are born (**Figure 3G**). The DZ B cell volumes exhibit a biphasic distribution, reflecting that these cells are actively engaged in growth and division (**Figure 3H**). In contrast, the LZ B cells exhibit a very narrow volume distribution consistent with the notion that they are mostly non-proliferating centrocytes.

##### 3.2.1.2 B cell death

As the GC B cell population grows, the number of cell deaths increases and then approaches a steady state, where the total death rate is about 1000 deaths per 600 *mcs* (**Figure 3I**). Although at the early time the DZ: LZ death rate ratio fluctuates dramatically as a result of randomness due to small numbers of cell deaths and DZ-LZ migration, the ratio stabilizes at about 2.5:1 at later time. The steady-state death turnover rate of the overall B cell population is slightly above 40% in 600 *mcs*, and the turnover rates in both zones are similar (**Figure 3J**). Cumulatively, there are nearly 70,000 cell deaths, among which 70% occurred in the DZ and 30% in the LZ (**Figure 3K**).

Further analysis showed that different types of cell deaths occur at different rates (**Figure 3L**). B cell death due to damaging BCR mutations occurs most often, comprising 65% of total deaths at steady state, followed by cell death due to no access to Tfh cells at 30%, while cell death for not being positively selected even after contacting Tfh cells (labelled as Neg selected) is only a small fraction of all death events (**Figure 3M**). When a B cell cannot access Tfh cells, positive selection decisions cannot be made in time and the B cell will die when the default death timer goes off. This type of death is only limited to B cells in the LZ initially, but as the GC population grows such that the space becomes more compact and thus crowded, such death also expands to the DZ when some of the B cells exiting the cell cycle do not have enough time to migrate through the densely populated DZ (**Figure 3N**); however, these deaths in the DZ are only a small fraction (**Figure 3O**).

#### 3.2.2 Affinity maturation and clonal dominance

**Figure 4:**
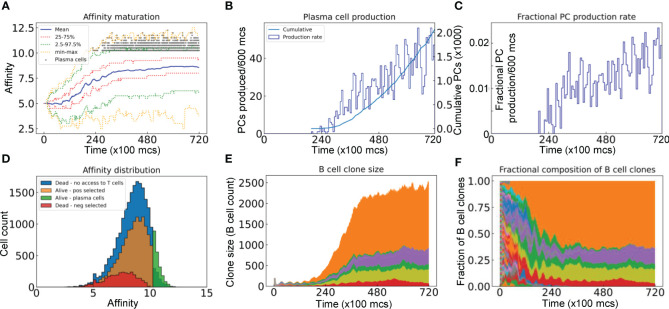
Evolution of B cell affinity maturation and clonal dominance. **(A)** Mean, interquartile, 2.5–97.5^th^ percentile, minimum and maximum BCR affinities of GC B cells as indicated. Gray dots denote the time and antibody affinities of PCs when they emerge. **(B)** Numbers of PCs produced every 600 *mcs* and cumulative numbers of PCs produced as indicated. **(C)** Fractions of B cells that differentiate into PCs in every 600 *mcs*. **(D)** Distributions of BCR/antibody affinities in B cells or PCs as indicated. **(E)** Evolution of B cell clone size as represented by the number of progeny B cells descending from each of the initial 200 clones. **(F)** Muller plot of evolution of the clonal fractions of the GC B cells. In **(E, F)**, each color represents a single clone of B cells.

We next characterized the evolution of the BCR antigen affinities in the simulated GC. With all the 200 seeder B cells starting with an intermediate BCR affinity of 5 in this simulation, their clonal affinities initially drift to both higher and lower levels (**Figure 4A**). However, the mean affinity increases progressively in a winding manner and then reaches a plateau at about 8.5. The variabilities of the BCR affinities, as defined by the 25–75% quantiles and 2.5–97.5% percentiles, also shift upward and then plateau along with the mean, despite that the affinities of some B cells reach as low as near 2 and as high as over 12 at times. PCs start to emerge shortly after 20,000 *mcs*, with >10 affinity levels (**Figure 4A**, 10 is defined as the threshold affinity to trigger terminal differentiation in the model). The production rate of PCs continues to increase albeit in a highly stochastic fashion and the total cumulative number of PCs produced at the end of GC reaches 2000 (**Figure 4B**). Only a tiny fraction of the B cell population becomes PCs in each 600 *mcs* time period, reaching as high as 2% at the end of simulation (**Figure 4C**). The PC antibody affinities range from 10 to 12.5, occupying the right tail of the overall affinity distribution (**Figure 4D**). For those B cells that are positively selected, their mean affinity is 8.28, which is higher than the mean affinity of 7.22 of those negatively selected cells. For those B cells that die due to no access to Tfh cells, their affinities cover a broader range on the high end, some of which reach 12.5. Among the initial 200 B cell clones, only 6 clones remain at the end (**Figure 4E**), among which one single clone dominates, comprising over 60% of the total B cells at 72,000 *mcs*, followed by two other clones each comprising about 12%, while the remaining 3 clones are much smaller (**Figure 4F**). Additional simulations showed that the fractions of dominant clones may vary for each GC – in some cases a single clone absolutely dominates the GC, occupying nearly 90% of the B cells (**Supplementary Figures S1A, S1B**), while in other cases the GC can be co-inhabited by several clones with no single dominant clone (**Supplementary Figures S1C, S1D**).

#### 3.2.3 Inter-zonal migration and LZ residence time

**Figure 5:**
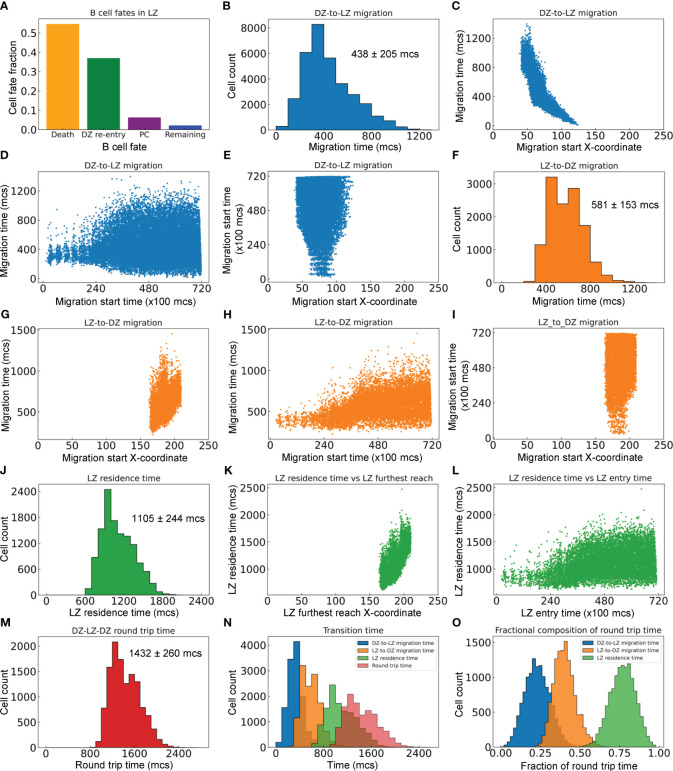
Quantitative analyses of inter-zonal migration. **(A)** Fractions of fates of B cells that have entered the LZ. **(B)** Distribution of DZ-to-LZ migration time (*T_DL_*) with mean ± std indicated. **(C)** Relationship between *T_DL_* and X-coordinate of DZ-to-LZ migration start location. **(D)** Relationship between *T_DL_* and DZ-to-LZ migration start time. **(E)** Relationship between DZ-to-LZ migration start time and location. **(F)** Distribution of LZ-to-DZ migration time (*T_LD_*) with mean ± std indicated. **(G)** Relationship between *T_LD_* and X-coordinate of LZ-to-DZ migration start location. **(H)** Relationship between *T_LD_* and LZ-to-DZ migration start time. **(I)** Relationship between LZ-to-DZ migration start time and location. **(J)** Distribution of LZ residence time (*T_LZ_*) with mean ± std indicated. **(K)** Relationship between *T_LZ_* and X-coordinate of LZ furthest reach of the B cells. **(L)** Relationship between T*_LZ_* and LZ entry time. **(M)** Distribution of DZ-LZ-DZ round trip time (*T_DLD_*) with mean ± std indicated. **(N)** Overlay of distributions of *T_DL_*, *T_LD_*, *T_LZ_*, and *T_DLD_*. **(O)** Distributions of *T_DL_*, *T_LD_*, and *T_LZ_* as fractions of *T_DLD_*.

We next characterized the statistics of B cell migrations between the DZ and LZ. For the GC simulation presented in **Figure 2**, there are a total of 33,440 B cells that enter from the DZ to the LZ, among which 54.6% die, 37.0% are positively selected and return to the DZ (thus making a full DZ-LZ-DZ round trip), 6.3% differentiate into PCs, and the rest remain in the LZ within the 72,000 *mcs* timeframe of the simulation (**Figure 5A**).

For the B cells that make a full DZ-LZ-DZ round trip, we calculated the durations they spend in different legs of the trip. The DZ-to-LZ migration time (*T_DL_*) is defined as the duration from the moment a B cell exits the cell cycle in the DZ and starts to migrate to the LZ to the moment the cell crosses the midline, i.e., the DZ/LZ border defined here. *T_DL_* follows a right-skewed distribution, with the median, mean, and standard deviation (std) at 390, 438, and 205 *mcs*, respectively (**Figure 5B**). *T_DL_* is inversely correlated with the X-coordinate where the DZ-to-LZ migration is started (**Figure 5C**), which is expected because migrations initiated further away from the DZ/LZ border will take a much longer time to complete than those initiated near the border. The variability of *T_DL_* increases as DZ-to-LZ migrations start at later times, broadening to both shorter and longer durations (**Figure 5D**). This increased variability can be attributed to the more spread-out X-coordinate of the migration start locations as the GC grows in size over time (**Figure 5E**).

Similarly, the LZ-to-DZ migration time (*T_LD_*) is defined as the duration from the moment a B cell is positively selected in the LZ and starts the DZ re-entry journey to the moment the cell crosses the midline. *T_LD_* also follows a right-skewed distribution, with the median, mean, and std at 570, 581, and 153 *mcs*, respectively (**Figure 5F**). *T_LD_* is positively correlated with the X-coordinate where the LZ-to-LD migration is started (**Figure 5G**), i.e., the migrations initiated further away from the DZ/LZ border take a longer time to complete than those initiated near the border. When the LZ-to-DZ migrations start at later times, the distribution of *T_LD_* broadens to the long side, without dropping further on the short side (**Figure 5H**). The broadening to longer durations can be attributed to the fact that as the GC grows, there are more LZ-to-DZ migrations that are initiated at locations in the LZ further away from the midline (**Figure 5I**). In comparison, the lower bound of the LZ-to-DZ migration time can be attributed to the locations of the Tfh cells in the LZ, where the ones nearest to the midline are at an X-coordinate of about 160 (**Figure 2A**), which is 35 pixels (µm) away from the midline.

The LZ residence time (*T_LZ_*), which contains *T_LD_*, is defined as the duration a B cell spends within the confine of the LZ before it reenters the DZ. Like the other two metrics, *T_DL_* and *T_LD_*, *T_LZ_* also follows a right-skewed distribution, with the median, mean, and std at 1065, 1105, and 244 *mcs* respectively (**Figure 5J**). *T_LZ_* is positively correlated with the X-coordinate of the furthest reach of the B cells in the LZ (**Figure 5K**), and broadens to longer times as the GC progresses (**Figure 5L**). There is no correlation between the LZ entry time and furthest reach in LZ (result not shown).

Lastly, we examined the DZ-LZ-DZ round trip time (*T_DLD_*) by summing *T_DL_* and *T_LZ_*. The median, mean, and std of the right-skewed distribution are 1395, 1432, and 260 *mcs*, respectively (**Figure 5M**). Examining the distributions of the components of *T_DLD_* indicated that on average a B cell spends the least amount of time migrating from DZ to LZ, followed by a 1.3-fold longer time migrating from LZ back to DZ (**Figure 5N**). A B cell spends a much longer time in LZ than in transition between the two zones, such that on average *T_LZ_* is about 1.9 and 2.5-fold longer than *T_DL_* and *T_LD_*, respectively. Overall, the round trip breaks down to 23% of the time spent on DZ-to-LZ migration and 77% on LZ residence of which 40% on LZ-to-DZ migration (**Figure 5O**).

#### 3.2.4 Proliferative burst and affinity

**Figure 6:**
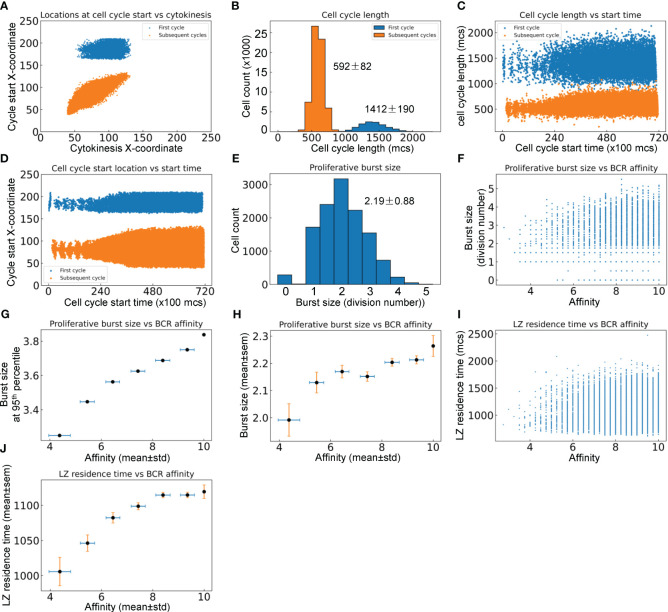
Quantitative analyses of proliferative bursts and BCR affinities of GC B cells. **(A)** Relationship between the X-coordinates of cell cycle start location and cytokinesis location for the first and subsequent cycles as indicated in a proliferative burst. **(B)** Distributions of cell cycle lengths of the first and subsequent cycles in a proliferative burst with mean ± std indicated. **(C)** Relationship between cell cycle length and start time of the first and subsequent cycles in a proliferative burst. **(D)** Relationship between cell cycle start location and start time of the first and subsequent cycles in a proliferative burst. **(E)** Distribution of proliferative burst size with mean ± std indicated. **(F)** Relationship between the proliferative burst size and affinity of DZ-returning B cells. **(G)** Association between the 95^th^ percentile proliferative burst size and mean affinity of DZ-returning B cells in each affinity bin as indicated. **(H)** Association between the mean proliferative burst size and mean affinity of DZ-returning B cells in each affinity bin as indicated. **(I)** Relationship between the LZ residence time and affinity of DZ-returning B cells. **(J)** Association between the mean LZ residence time and mean affinity of DZ-returning B cells in each affinity bin as indicated.

B cells positively selected in the LZ initiate their proliferative burst by entering S phase while migrating back toward the DZ (12, 66), and complete the first division of the proliferative burst primarily in the DZ. Since *T_LD_* = 581 ± 153 *mcs*, a time delay of 800 *mcs* for cell growth in the first cell cycle was introduced in the model to ensure that cytokinesis does not occur before B cells reach the DZ. Simulations confirmed that this is indeed the case for the first cell cycles of proliferative bursts of nearly all DZ-returning B cells, while all subsequent cycles in the bursts are started and completed within the DZ (**Figure 6A**). In a proliferative burst, the length of the first cell cycle is 1412 ± 190 and those of subsequent cycles are 592 ± 82 *mcs* respectively (**Figure 6B**). Putting these numbers into perspective, the subsequent cycles have an average length equivalent to nearly 6 hours. The variability of cell cycle length increases as the GC progresses, particularly on the long side (**Figure 6C**), suggesting that in a more crowded space, more time is needed to allow “pixel copy” to occur in the CompuCell3D environment in order for cells to grow to the pre-cytokinesis volume. The X-coordinate locations at which cell cycles are initiated expand, especially for subsequent cycles in a proliferative burst (**Figure 6D**). The average proliferative burst size, i.e., the number of cell divisions in each burst, is 2.19 ± 0.88 (**Figure 6E**). While the majority of B cells that return to the DZ can mount a burst of 2 divisions, some go on to divide up to 5 times. Examining the relationship between the affinity of each DZ-returning B cell and its burst size (**Figure 6F**) revealed that while a high affinity does not guarantee a large burst size (due to the randomly produced damaging BCR mutations that would kill the daughter cells and thus terminate the burst prematurely), the affinity appears to be positively correlated with the highest achievable burst size. When the affinities are binned into different intervals: <5, 5–6, 6–7, 7–8, 8–9, and >=10, the burst size at the 95^th^ percentile in each bin, which are likely the proliferations that contribute to the dominating B cells in the GC eventually, is positively associated with the affinity (**Figure 6G**). The mean burst size in each affinity bin also exhibits a positive albeit less strong relationship with the affinity (**Figure 6H**). Higher affinities correlate with longer LZ residence time (**Figures 6I, J**), which is likely a result of association rather than causality, since improvement in affinity and GC population growth occur simultaneously over time, and a denser GC population at a later time tends to result in a longer navigating time through the LZ due to crowding (**Figure 5L**).

### 3.3 Molecular responses of B cells and their relationships with cellular phenotypes

**Figure 7:**
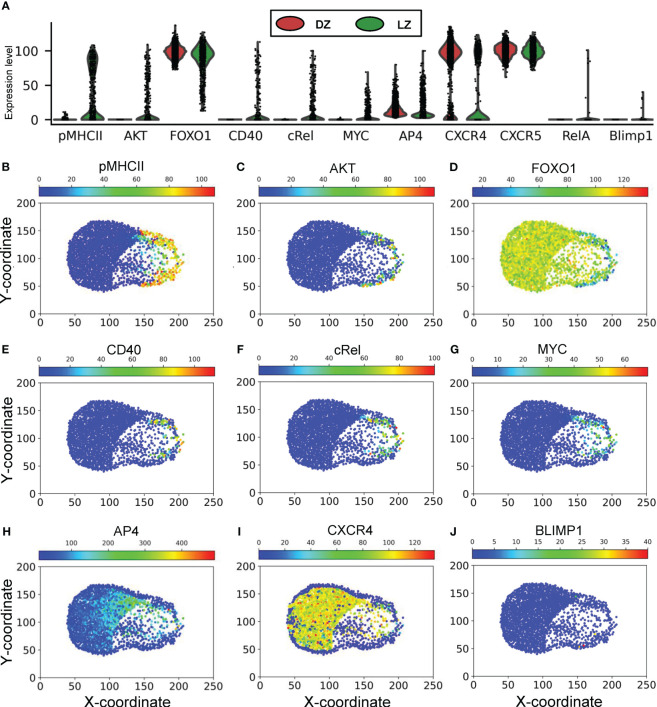
Molecular response profiles of GC B cells. **(A)** Violin plots of expression/activity levels of signaling molecules in DZ and LZ B cells as indicated at 70,000 *mcs*. **(B–J)** Simulated “immunohistochemistry” staining of signaling molecules as indicated in GC B cells at 70,000 *mcs*.

The above patterns of phenotypical behaviors of B cells are underpinned by the molecular network operating in each B cell responding to extracellular signaling cues in the GC, including chemoattractants CXCL12 and 13, BCR-mediated antigen signaling by engaging FDCs, and CD40 signaling by engaging Tfh cells (**Figure 1**). Examining the steady-state GC population at 700,000 *mcs* revealed that the expression or activity levels of the signaling molecules and genes in the 2352 B cells in the DZ and LZ are highly heterogeneous (**Figure 7A**). While there is no pMHCII expression in the DZ, a large fraction of B cells in the LZ expresses high pMHCII levels (**Figure 7B**), and those expressing low pMHCII levels are B cells that either have just entered the LZ or are en route returning to the DZ with pMHCII being downregulated. A small fraction of B cells in the LZ exhibits high AKT levels as they interact with FDCs (**Figure 7C**), which causes downregulation of FOXO1 (**Figure 7D**). Signaling downstream of CD40 signaling are cRel and MYC, which are active in a small fraction of B cells in the LZ (**Figures 7E-G**). MYC activity induces AP4, which remains upregulated for an extended period of time, even after the B cells have returned to the DZ and MYC has been downregulated (**Figure 7H**), to sustain proliferative bursts. The majority of B cells in the DZ express CXCR4 and the small fraction with CXCR4 downregulated are B cells that have exited the cell cycle and are about to migrate to the LZ (**Figure 7I**). There is no difference in CXCR5 expression in B cells between the two zones (**Figure 7A**). A few cells in the LZ exhibit high RelA (**Figure 7A**) and BLIMP1 (**Figure 7J**) expression representing emerging PCs. The correlations between select pairs of the signaling molecules are shown in **Supplementary Figure S2**. There is a strong negative correlation between FOXO1 and AKT (**Supplementary Figure S2A**), positive correlations between cRel and CD40 (**Supplementary Figure S2C**), MYC and CD40 (**Supplementary Figure S2D**), MYC and cRel (**Supplementary Figure S2E**), and AP4 and MYC (**Supplementary Figure S2F**) in B cells in the LZ, while CXCR4 is only expressed in high FOXO1-expressing B cells (**Supplementary Figure S2B**).

**Figure 8:**
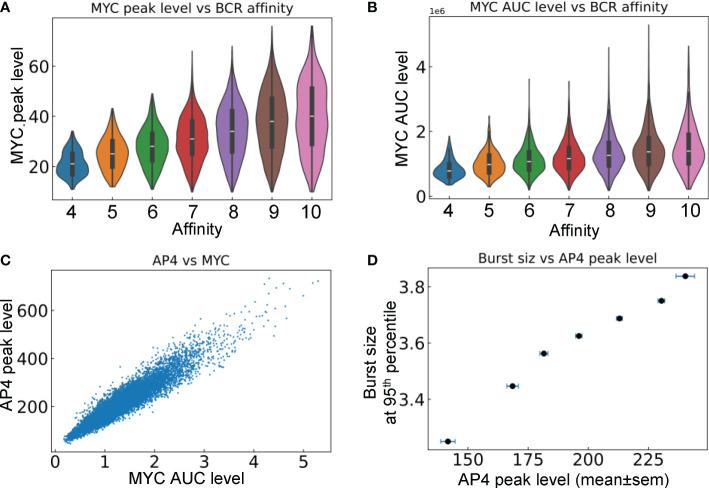
Relationships between signaling molecules and phenotypical behaviors of DZ-returning B cells. **(A)** Violin plots of MYC peak levels in different affinity bins. **(B)** Violin plots of MYC AUC level in different affinity bins. **(C)** Correlation between AP4 peak level and MYC AUC level. **(D)** Correlation between the 95^th^ percentile proliferative burst size and mean AP4 peak level in each affinity bin.

We next examined the relationship between the signaling molecules and phenotypical behaviors of B cells that are positively selected in the LZ and return to the DZ. Both the peak level (**Figure 8A**) and duration of MYC expression (**Figure 8B**), which is quantified by the area under the curve (AUC), are positively correlated with the BCR affinity of the cells. The correlation indicates that the strength of the affinity-dependent BCR signaling, which downregulates FOXO1 and upregulates pMHCII to enable Tfh-dependent CD40 signaling, is quantitatively transmitted to MYC. Although MYC is only transiently expressed in the positively selected B cells in the LZ, its encoding of affinity is relayed to AP4. By integrating the MYC signal, AP4, which has a longer half-life than MYC, can be activated for a much longer time as B cells migrate back to the DZ. Its peak level is positively correlated with the AUC of MYC in DZ-returning B cells (**Figure 8C**). The 95^th^ percentile burst size is positively correlated with the mean AP4 peak level in each affinity bin (**Figure 8D**).

**Figure 9:**
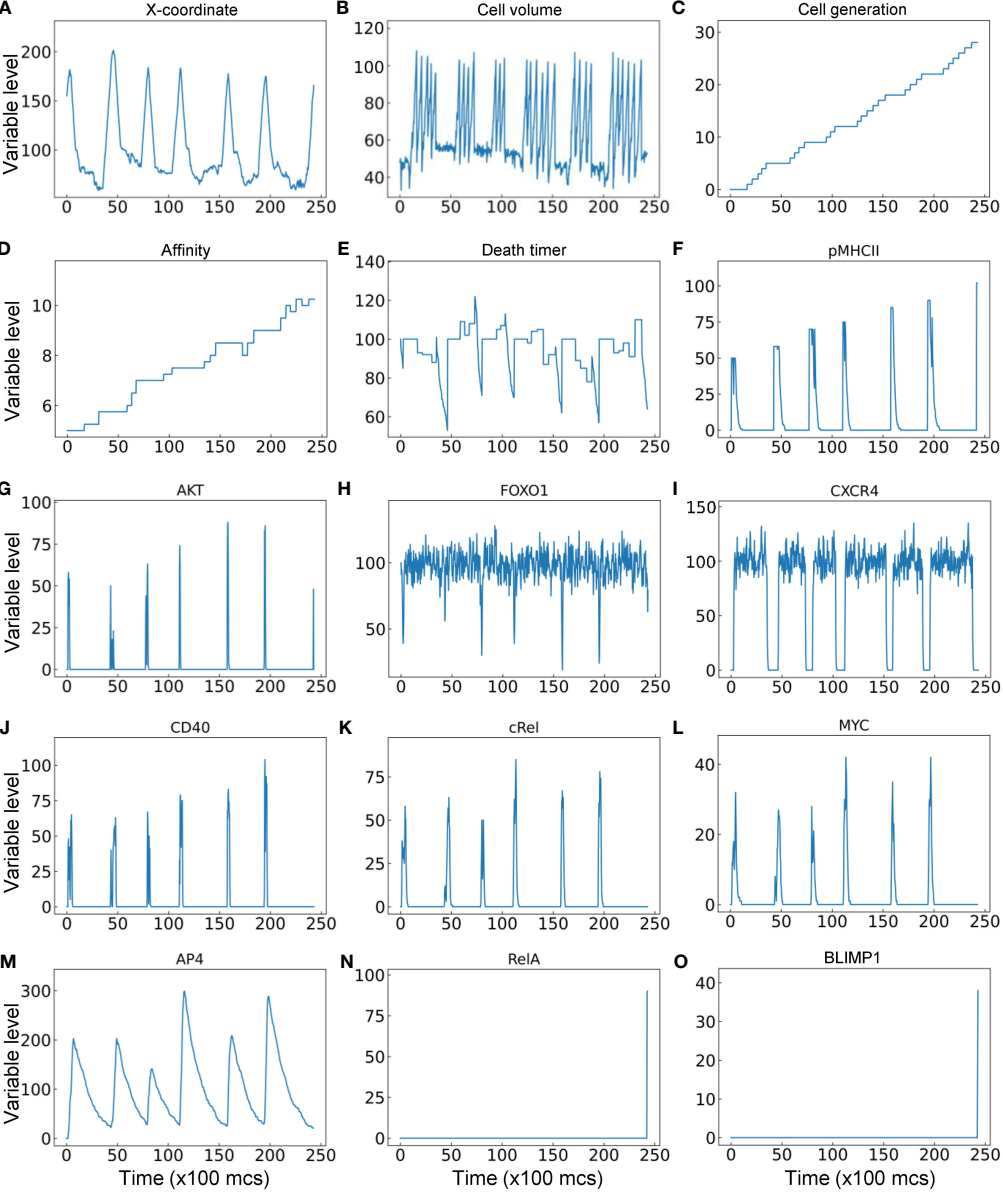
Trajectory of a single B cell lineage branch that successfully makes it to a PC with cellular **(A–E)** and molecular **(F–O)** variables as indicated.

Lastly, the trajectory of a representative single B cell lineage branch that successfully makes it to a PC is presented (**Figure 9**). As the seeding B cell and its progeny cycle between the DZ and LZ (**Figure 9A**), multiple cell divisions (between 3–6 divisions) occur in each proliferative burst (**Figure 9B**) with increasing cell generation (**Figure 9C**). In this particular simulation result, nearly every cell division results in an increase in BCR affinity despite a few occasions when the affinity decreases (**Figure 9D**). Each time the B cell starts the trip to the LZ the countdown of the death timer is initiated, but in this case it never drops below the predefined death threshold of 50 before the cell is rescued by positive selection where the death timer is reset (**Figure 9E**). Every time the B cell moves into the LZ, pMHCII is re-expressed proportionally to the antigen-specific affinity after encounter with FDCs that triggers BCR signaling (**Figure 9F**). BCR signaling transiently activates AKT (**Figure 9G**) which downregulates FOXO1 transiently (**Figure 9H**). Commitment to cell cycle after the B cell is positively selected allows upregulation of CXCR4 by FOXO1, which drives the B cell to return to the DZ, and CXCR4 is downregulated after the B cell exits the last cell cycle of a proliferative burst (**Figure 9I**), which allows the cell to migrate to the LZ due to constitutively expressed CXCR5 (not shown). In the LZ, encounter with Tfh cells triggers transient activation of the CD40-cRel-MYC axis in the presence of downregulation of FOXO1 (**Figures 9J-L**). By integrating the MYC signal, AP4 is upregulated for an extended period of time, which lasts well into the DZ (**Figure 9M**) to sustain the proliferative bursts. The proliferative bursts terminate when AP4 drops below a predefined threshold of 50. When the affinity increases past a predefined threshold of 10, the BCR signaling triggers RelA activation in a switch-like manner (**Figure 9N**), which in turn activates BLIMP1 that drives the B cell to terminally differentiate into a PC (**Figure 9O**). A representative result of a B cell lineage that ends in death in the DZ due to damaging BCR mutations is shown in **Supplementary Figures S3A-S3C**, where at the time the damaging mutation occurs the affinity drops to “-1” as an indication, and caspase 3 is upregulated to trigger apoptosis. A representative result of a B cell lineage that ends in death in the LZ because of not being positively selected is shown in **Supplementary Figures S3D-S3F**, where the death timer dips below the threshold of 50 thus triggering cell death.

## 4 Discussion

In the present study, we developed a multiscale spatial modeling framework for the GC in the CompuCell3D simulation platform. By integrating interactions across molecular, cellular, and tissue scales, the model captures key hallmark GC events, including cyclic migration of B cells between the DZ and LZ, proliferative burst, SHM, deaths due to damaging BCR mutation, positive selection, timed cell death, affinity maturation, clonal expansion and dominance, and loss of clonal diversity. As detailed in sections below, the model recapitulates a number of mature GC characteristics that are quantitatively consistent with the literature, including DZ: LZ B cell ratio (67), scaled number of B cells (68), percentage death rate in the DZ and LZ (15), fraction of GC B cell differentiating into PCs (69–71), fraction of LZ B cells that return to the DZ (7, 12, 35), the DZ-LZ migration time (12, 72), and the average and range of proliferative burst size in the DZ (35, 66, 73, 74). The cellular behaviors are driven by simulating an underlying molecular network in individual B cells responding to BCR and CD40 activation via interacting with FDCs and Tfh cells, respectively. The molecular outputs of the network include CXCR4 driving LZ-to-DZ chemotaxis, MYC and AP4 driving cell cycles, and caspase 3 driving apoptosis. While this multiscale model can be further tuned and elaborated to study diverse variables and conditions regulating GC outcomes, exploring these possibilities is beyond the scope of the present study. We focused on demonstrating the capability of the modeling framework by presenting a simulated GC that leads to affinity maturation, with qualitative and to some extent quantitative results that are commensurate with the primary literature.

### 4.1 GC B cell birth, death, and population dynamics

Emerging GCs are seeded with up to a few hundred B cell clones (68, 75). Starting with 200 B cells, our GC simulation showed that the DZ: LZ ratio of the numbers of B cells oscillates at early time points (**Figure 3A**). This occurs because there are not many B cells at this stage of the GC and these cells tend to migrate in sync between the two zones. As the GC B cell population approaches a steady state, the DZ: LZ ratio converges to a constant value, nearly 2.6:1, in our simulation, which is comparable to the 2.15~2.2:1 ratio observed in both mouse and human GCs (67). The total number of B cells in the simulation can reach about 2500. Given that the modeled GC space contains only 11 pixels (equivalent to 11 µm) along the Z-dimension to keep the simulation time tractable, we expect that when scaling up by 2–5 times to mimic the actual GC thickness of 20–50 µm (76), the peak number of B cells will reach thousands to over 10,000, consistent with the estimated B cell counts in real GCs (68).

Our simulation showed that new B cells are born in the DZ, while cell deaths occur in both the DZ and LZ at an overall rate that eventually matches the birth rate as the GC approaches the steady state. Although the absolute number of deaths in the DZ is more than twice that in the LZ (**Figure 3I**), the percentage death rates are comparable, at about 40–43% per 600 *mcs* in both zones (**Figure 3J**). These numbers are concordant with the nearly 50% death rate per 6 hours reported for the GCs in Peyer’s patches in mice and GCs in mice immunized with 4-hydroxy-3-nitrophenylacetyl (NP)-conjugated ovalbumin (NP-OVA) or HIV-1 envelope antigen GT1.1 (15). The steady-state death turnover rate of 50% per 6 hours is expected given that GC B cells proliferate with a cell-cycle length of 4–6 hours (10, 24) and in our model the cell volume doubling time of proliferating B cells is parameterized at 500 *mcs*. The apoptotic deaths in the DZ are believed to occur in the late G1 phase, triggered by AID-induced BCR-damaging mutations during transcription, including stop codons, insertions and deletions in the Ig sequences, such that the synthesized BCR proteins fail to properly fold and be expressed on the cell surface (14, 15).

In the LZ, the default fate of B cells is apoptosis if not positively selected, regardless of their BCR affinity, and the apoptosis will occur when the death timer goes off, which is initiated after the B cells exit the cell cycle in the DZ (15, 77). Our simulations showed that only a small fraction of deaths in the LZ result from not being positively selected after contact with FDCs and Tfh cells, while the majority of the deaths are due to no access to Tfh cells at all (**Figure 3M**). This result recapitulates the current notion that the availability of Tfh cells is the limiting factor, not the competition for antigen, for positive selection (12, 63–65). Interestingly, our simulations also predicted that at the advanced GC stage, a small fraction of cell deaths in the DZ may also result from the lack of access to Tfh when these cells do not have enough time to migrate through a densely populated DZ to reach the LZ (**Figures 3N, O**) and some of these dead cells could be of high-affinity. Whether deaths of such nature occur *in vivo* and to what extent remain to be tested experimentally using techniques including intravital imaging of apoptotic GC B cells, single-cell cloning of *Ig* genes from dying B cells, and recombinant antibody expression as in (15).

The dynamics of the GC B cell population is determined primarily by the cell birth rate and death rate. The loss of B cells as a result of GC exit as memory and PCs is expected to be negligible because they only account for <3% of the GC B cell fates (69–71). Our simulation result is consistent with this estimate: PCs emerge at a fraction of only as high as 2% of the B cell population towards the end of the simulated GC (**Figure 4C**). For a GC B cell population to grow or to avoid population collapse, the average proliferation rate has to be higher than the death rate. Absent any limiting factors, the cell population dynamics in the GC may operate as a positive feedback system, producing bistability of two alternative outcomes – expansion or regression – as predicted by previous mathematical models (78). In the expansion mode, as the overall affinities increase, the probability of cell death in the LZ owing to lack of positive selection decreases, thus more B cells will return to the DZ and proliferate with a larger burst size there, which results in a higher birth rate of progeny cells with potentially even higher affinities returning to the LZ, and the cycle repeats leading to GC B cell expansion. In the regression mode, the positive feedback works in the opposite direction, where lower affinities can lead to fewer B cells positively selected in the LZ and smaller burst size in the DZ, which eventually leads to GC regression. This all-or-none type of GC outcome is consistent with the population bottleneck proposition (79) and suggests that there could be an initial affinity threshold condition for those activated B cells that seed a GC, below which a tangible GC is unlikely to emerge and above which a GC will likely emerge and grow. This suggests that it may take B cells of some intermediate affinity to initiate a GC to produce optimal humoral immune outcomes, i.e., high production of high-affinity PCs. A GC starting with B cells of too low affinity will likely abort prematurely as argued above, while a GC starting with high-affinity B cells may grow but only to a small size before some B cells hit the affinity threshold that triggers terminal differentiation to PCs. This may also explain the observation that affinity selection for memory B cells is less stringent, and they are often formed and then exit the GC when their affinities are still at low or intermediate levels, while the PCs are in general of high affinity (71, 80–83). Upon secondary infection or booster immunization whether the recalled memory B cells directly differentiate to PCs or have to go through GCR again can be determined by many factors (84, 85), and it is likely that their BCR affinity may play a role in this regard.

When a GC reaches a certain size, its growth could be restricted by multiple factors, before other GC-shutoff mechanisms such as antigen depletion and antibody feedback kick in. In the present study, we showed that the availability of Tfh cells is a limiting factor, where a higher fraction of B cells in the LZ die because of lack of access to Tfh cells, thus increasing the overall death probability which balances out the increasing birth rate due to improved antigen affinity. That B cells become PCs once their affinities reach a threshold also helps the GC B cell population to reach an equilibrium by curbing further increase in the number of higher-affinity B cells and thus attenuating the positive feedback mechanism described above. Another limiting factor not considered in the present study is the limited nutritional and energetic resources that could restrict B cell proliferation once the GC has grown to a mature size (68).

To grow or maintain a GC the overall damaging BCR mutation-induced cell death probability cannot be higher than 50% for each cell division. Actually it has to be much lower than 50% because not all but only a small fraction of B cells arriving in the LZ are positively selected and return to DZ. In our model, we encoded a DZ death probability of 30% for each of the two daughter cells born from a cell division, which leads to a probability of 49% (0.7*0.7) to double the number of B cells after each division, of 42% (2*0.3*0.7) to keep the number of B cells constant, and of 9% (0.3*0.3) to eliminate the proliferating B cell. The DZ re-entry probability was estimated to be between 10–30% through mathematical modeling and analysis of experimental data (7, 12, 35). These values suggest that absent any DZ death, the average proliferative burst size has to be greater than 1.73–3.32 divisions to grow a GC. When taking into consideration DZ death, which is very significant, on par with LZ death (15), the average burst size has to be much higher. The DZ re-entry probability depends on the BCR antigen affinity, thus it is likely that at the early stage of GC the re-entry probability is low and at the advanced stage it is high. In our model, the positive selection and thus DZ re-entry probability is set to be proportional to pMHCII, such that when the affinity is intermediate at 5, the probability is 50% and when the affinity approaches 10 or higher, the DZ re-entry probability is 100%. However, the overall DZ re-entry fraction is only 36% in our simulation (**Figure 5A**), which can be attributed in part to the inaccessibility to Tfh cells. A re-entry fraction of 36% requires at least a burst size of 1.47 to grow the GC in the absence of DZ death. With a DZ death probability of 30% for each new born B cell, our model has an average burst size of 2.2 (**Figure 6E**), which is in general agreement with the estimated average of 2 divisions (35, 66, 73) or 3 divisions per burst (74) in the literature.

### 4.2 Affinity maturation, proliferative burst, clonal expansion and dominance

While the average proliferative burst size is 2–3, each burst can vary between 1–6 divisions (35, 66, 73, 74). This range is quantitatively captured in the burst size distribution produced by our simulation (**Figure 6E**). The right-tailed burst size distribution could result intrinsically and in part from the probabilistic damaging mutation-induced cell death after each cell division, which increases the chance of short bursts but limits the highest attainable number of divisions in a proliferative burst even for high-affinity B cells. GC B cells positively selected are guaranteed to divide once, while the number of additional divisions or the burst size is directly proportional to the amount of antigen captured by B cells from FDCs and presented to Tfh cells (66, 86). Our model reproduces this positive association (**Figures 6G, H**). Moreover, because of potential premature termination of a proliferative burst induced by damaging mutation, the affinity appears to be better correlated with the top attainable burst size than the average burst size.

The translation of BCR antigen affinity into burst size is mediated molecularly by two key transcription factors: MYC and one of its target genes, AP4. Because of the transient nature of B cell interactions with FDCs and Tfh cells (65) and the short half-life of MYC (77), MYC is only transiently expressed in a small fraction of LZ B cells (49, 87). The MYC expression level is in direct proportion to the amount of antigen captured and dictates the proliferative burst size (86). While MYC can initiate cell growth and cell cycle by driving LZ B cells into the S phase, its lasting effect on cell proliferation is mediated by AP4, which is induced by MYC in a delayed fashion in positively selected GC B cells and is sustained after the B cells re-enter the DZ (50). Our model recapitulates the spatiotemporal dynamics of MYC by showing transient MYC expression in LZ B cells, its positive correlation with BCR antigen affinity, and sustained AP4 expression.

Our model recapitulates a typical affinity maturation process along with GC growth (**Figure 4A**). The progressive increase in the mean affinity of the GC B cell population is not because all or the majority of the seeding B cell clones improve their affinities uniformly. Rather, in most cases, the mature GC B cell population is dominated by progenies of one or a few of the initial 200 seeding clones (**Figure 4F**; **Supplementary Figures S1B, S1D**). This simulation result of terminal clonal dominance is consistent with the premise that GCs mature oligo-clonally (88, 89). More recently, using multiphoton microscopy and sequencing, Tas and colleagues further revealed that a GC can start with tens to hundreds of distinct B cell clones but loses the clonal diversity over time, converging to one or a few parallelly expanding clones (75). The single dominant clone can constitute 10–100% of the final GC B cell population. They further showed that clonal dominance can be achieved through neutral competition, due to stochastic effect, even when all seeding B cells have equal affinity and cannot undergo SHM, a finding that can be explored with our model in the future.

### 4.3 Inter-zonal migration

Beltman et al. analyzed time-lapsing imaging data of GCs and revealed that B cells move at a net speed of 0.2–0.3 µm/min toward the LZ to produce a DZ-to-LZ migration time of a few hours (72). In our simulation, the DZ-to-LZ migration time is about 438 ± 205 *mcs* (**Figure 5B**), consistent with that estimated by Beltman. The LZ-to-DZ migration time in our simulation is 581± 205 *mcs* (**Figure 5F**) which is about 33% longer than the DZ-to-LZ migration time. The longer time is consistent with the experimental observations (12), and could be attributed to the fact that B cells returning to the DZ have to move against the much heavier incoming traffic of B cells migrating from DZ to LZ. The overall LZ residence time is 1105 ± 244 *mcs* (**Figure 5J**). These travel times can be tuned by varying the positions and distributions of CRCs and FDCs/Tfh cells in the DZ and LZ respectively, the CXCL12 and 13 gradients, and the chemotaxis strength parameters in the model.

### 4.4 Existing computational GC models and improvements by our modeling framework

Many mathematical GC models have been developed in the past two decades using a variety of computational approaches, including deterministic, stochastic, agent-based, and hybrid ones. Focusing on various aspects of the GCR and simulating at various biological scales, these models have greatly aided our understanding of this long known phenomenon by investigating possible modes of mechanisms of GC dynamics (34, 35, 72, 90–94), exploring optimal design of vaccination schemes (95–99), and predicting GC-associated disease outcomes (100). While these models focused on the complex processes of affinity maturation and B cell population dynamics, rarely were molecular networks included to drive the B cell behaviors and GC evolution. Quantitative multiscale mathematical models of GC dynamics have been proposed as predictive frameworks to translate basic immunological knowledge to practical challenges (36, 101). In recent years, modeling efforts in this direction have emerged. For example, Merino Tejero and colleagues have developed multiscale GC models integrating molecular and cellular responses (37, 39), by combining the agent-based model developed in (35) and ODE-based gene regulatory network model comprising BCL6, IRF4, and BLIMP1 developed in (102). The early model was used to study the role of affinity-based CD40 signaling and asymmetric B cell division in temporal switch from memory B cell to PC differentiation and DZ-to-LZ ratio. Lately, the model was adapted to examine the oncogenic effects of genetic alteration of the above key transcription factors on GC-originated diffuse large B cell lymphoma (38). More recently, the model was used to explore the relationship between clonal abundance and affinity as well as affinity variability in intraclonal B cells, with an attempt to make sense of repertoire sequencing data (103).

In comparison, the multiscale spatial GC modeling framework we developed here in the CompuCell3D platform further integrates across the molecular, cellular, and tissue scales and offers several improvements. The framework allows the molecular network to drive multiple cellular behaviors, including B cell growth, division, chemotaxis, survival/death, and PC differentiation, which in turn collectively drive GC tissue pattern formation; reciprocally, the cell-to-cell interactions between B cells and FDCs and Tfh cells drive the responses of the molecular signaling network in B cells. Novel cross-scale strengths include cell cycle and FOXO1-dependent CXCR4 expression driving DZ-reentry chemotaxis, MYC and AP4-dependent cell growth and division burst, and RelA and BLIMP1-dependent PC differentiation. As more molecular species are added to the network, additional cross-scale integrations will become available. Because the simulated B cells comprise multiple pixels, the model allows recapitulation of B cell morphology during chemotaxis and volume growth during cell cycle as well as better mimicking of cell-cell interactions. For future iterations of the model, the CompuCell3D platform can readily include paracrine signaling by ILs and other cytokines secreted by B cells, Tfh cells, and FDCs. Last but not least, with the modular plugins and systems biology markup language (SBML) support, the CompuCell3D platform allows a more structured construction of the GC model that will facilitate future model sharing and integration.

### 4.5 Limitations and future iterations

As an initial effort to establish the multiscale GC modeling framework on the CompuCell3D platform, we simulated the GC to the mature stage where the B cell population approaches a steady state. While persistent GCs can exist for months or years under certain viral infections (104–106), and in the Peyer’s patch for mucosal immunity (107), in most other infection or immunization scenarios, GCs eventually regress in 3–4 weeks with mechanisms still incompletely understood. Some GC terminations may be because the antigens stored in FDCs are depleted, the signaling nature of Tfh cells and FDCs has changed, or antibodies produced by the departed PCs circulate back into the GC and block antigen presentation (92, 108, 109). To demonstrate that our modeling framework is capable of self-termination, we simulated a scenario of gradual FDC antigen depletion after the GC matures. The simulation clearly showed that the GC eventually regresses, yet preserving many characteristics seen had it persisted, including DZ: LZ B cell ratio, affinity maturation, and clonal dominance (**Supplementary Figure S4**).

In the current study, we presented the simulations where all GC seeder B cells have the same initial affinity. Randomizing the initial affinity between 3–7 uniformly produced a similar result on GC B cell population growth, affinity maturation, and clonal dominance (**Supplementary Figure S5**). Future investigations may explore the outcomes of more complex initial affinity compositions. In addition, it will be interesting to use the model to explore alternative hypotheses for mechanisms underpinning the clonal replacement phenomenon observed in long-lived GCs, as triggered by influenza virus or SARS-CoV-2 infection, where the founder B cell clones of high SHM loads and high affinity in mature GCs are gradually replaced by newcomer B cells that are not necessarily antigen-specific (110). In the current model, memory B cells are not included as a cell fate option. Since like PCs, memory B cells only constitute a very small fraction of the GC B cells fates (109), its exclusion is not expected to affect the overall model behavior. Nonetheless, future iterations of the GC model will include the self-termination and memory B cell formation as driven by the IRF4-BCL6-BLIMP1 network (102), and if needed, CSR, which is believed to occur primarily during pre-GC formation (56).

A small caveat of the current model, as currently implemented on the CompuCell3D platform, is that when the cell density is too high such that the DZ is packed, there is a small chance that some dividing B cells vanish in the DZ when their volumes are too small (as represented by the small left tail of the DZ B cell volume distribution in **Figure 3H**). This issue can be prevented in future iterations by imposing a limiting resource for cell growth and division to control DZ cell density. B cells are highly mobile in both the DZ and LZ, with a mobility pattern observing persistent random walk (65, 72, 111). While we did not analyze the B cell mobility in this regard, the CompuCell3D platform, which is based on the Cellular Potts Model that follows the Boltzmann law (58), is capable of simulating persistent random walk (112). In our case, the *temperature* parameter and local CXCL12 and 13 distribution patterns in the LZ and DZ can be optimized, along with reducing the directional chemotactic forces, to help accentuate the random-walk effect. As indicated above, it was previously showed that the persistent random walk of B cells could be an emergent behavior of B cells in a crowded GC environment (113), thus it will be interesting to inspect our GC model in this regard.

In the current model, the two daughter cells split the parent cell’s volume by following a lognormal distribution. While this approach implemented some degree of cell division asymmetry, in future iterations asymmetrical cell divisions can be better implemented based on experimental studies which showed asymmetrical segregation of pMHCII among the two daughter cells, which plays a role in B cell fate decision-making (114), and was implemented in recent models (37, 39). In the current model the length of each cell cycle in a proliferative burst is targeted as a constant. It has been shown that the S phase, which constitutes the major portion of the cell cycles of proliferating B cells, can be shortened by regulating replication fork progression, while the relative order of replication origin activation is preserved (74). The degree of S-phase shortening depends on the interaction strength of B cells with Tfh cells which in turn depends on BCR antigen affinity. Therefore, positively selected high-affinity GC B cells, upon returning to the DZ, will proliferate not only with a larger burst size but also with accelerated cell cycles. This could be a mechanism to compensate for the tendency of longer DZ residence time of high-affinity B cells due to more cell divisions such that they can return to the LZ sooner. Future iterations of the model may consider incorporating affinity-dependent cell cycle shortening.

The molecular network model running in each B cell uses the Gillespie’s stochastic simulation algorithm, with some of the molecular switching actions implemented as hybrid, rule-based events. For simplicity and parsimony, several genes known to participate in GCR and B cell terminal differentiation are not included in the current implementation of the GC model, including BCL6, IRF4, BACH2, and PAX5. BCL6 is upregulated in antigen-engaged B cells in the early stage of GC initiation, before these cells migrate back into the intrafollicular space and cluster in the GC (115). There does not appear to be cyclic BCL6 expression between the DZ and LZ. Since our current model starts with B cells seeding the GC, not including the initial interactions at the T/B border, the absence of BCL6 in the molecular network should not affect the simulation results. Likewise, IRF4, BACH2, and PAX5 do not seem to exhibit cyclic expression patterns during GCR despite that they are involved in either the initiation of GC, AID induction, or terminal differentiation of B cells to PCs or memory cells (116–118). Therefore, by not including them the current model is made simpler. Nonetheless, BCL6, IRF4, BACH2, BLIMP1, and PAX5 are known to form coupled positive or double-negative feedback loops underpinning multistability-based binary decision-making in B cells (119, 120). In future iterations, the hybrid stochastic and rule-based approach of molecular network simulation can be updated by implementing relevant intracellular feedback circuits comprising these transcription factors to enable bi- and multistability-based decision-making on B cell fates. To this end, single-cell RNA sequencing data of GC cells can be integrated in to the molecular network model to parameterize the molecular abundance of the gene transcripts (70).

There are a number of other considerations that could be potentially added in future iterations as well. Once seeded, a GC reaches peak volume in 3–4 days (121, 122), resulting from an initial clonal expansion without SHM and competitive selection (113, 123–125). Future models should consider implementing this process to bring the GC kinetics to more closely align with such rates of B cell accumulation. Paracrine signals mediated by ILs and other cytokines secreted by B cells, Tfh cells, and FDCs may be considered to better recapitulate the tissue-level molecular signaling milieu in the GC. Mobility of Tfh cells, and their intracellular signal transduction and gen regulatory network can be included. The shapes and locations of residential CRCs and FDCs and as a result the spatial patterns of CXCL12 and 13 gradients can be fine-tuned according to immunohistochemistry data, which may ultimately affect the morphology and polarity of GCs.

### 4.6 Conclusions

In conclusion, we have developed a multiscale spatial computational modeling framework for GC simulation in CompuCell3D. The current model is capable of recapitulating GC features that are both qualitatively and to some extent quantitatively consistent with the literature. Given the complexity of GCR, simulations in such a modeling framework can help investigate a range of research questions on this hallmark event of high-affinity antibody production in response to viral infection and vaccination. Upon further extension and refinement, this open-source modeling framework may also help investigate autoimmunity and lymphoma when the GC goes awry due to genetic or environmental disruptions. Lastly, the GC modeling framework may also be utilized towards building digital twins of the human immune system for precision medicine (126).

## Data availability statement

The original contributions presented in the study are included in the article/**Supplementary Material**. Further inquiries can be directed to the corresponding author.

## Author contributions

DM: Formal analysis, Investigation, Methodology, Software, Writing – original draft, Writing – review & editing, Visualization. CS: Conceptualization, Funding acquisition, Investigation, Writing – review & editing. NK: Conceptualization, Funding acquisition, Investigation, Writing – review & editing. QZ: Conceptualization, Formal analysis, Funding acquisition, Investigation, Methodology, Project administration, Software, Supervision, Validation, Writing – original draft, Writing – review & editing.
