# Supplementary material for: A multiscale spatial modeling framework for the germinal center response
Source: Front Immunol. 2024 May 30;15:1377303. doi: 10.3389/fimmu.2024.1377303 (PMC11179717; doi:10.3389/fimmu.2024.1377303)
Supplement: Supplementary file 1 [file DataSheet_1.pdf]

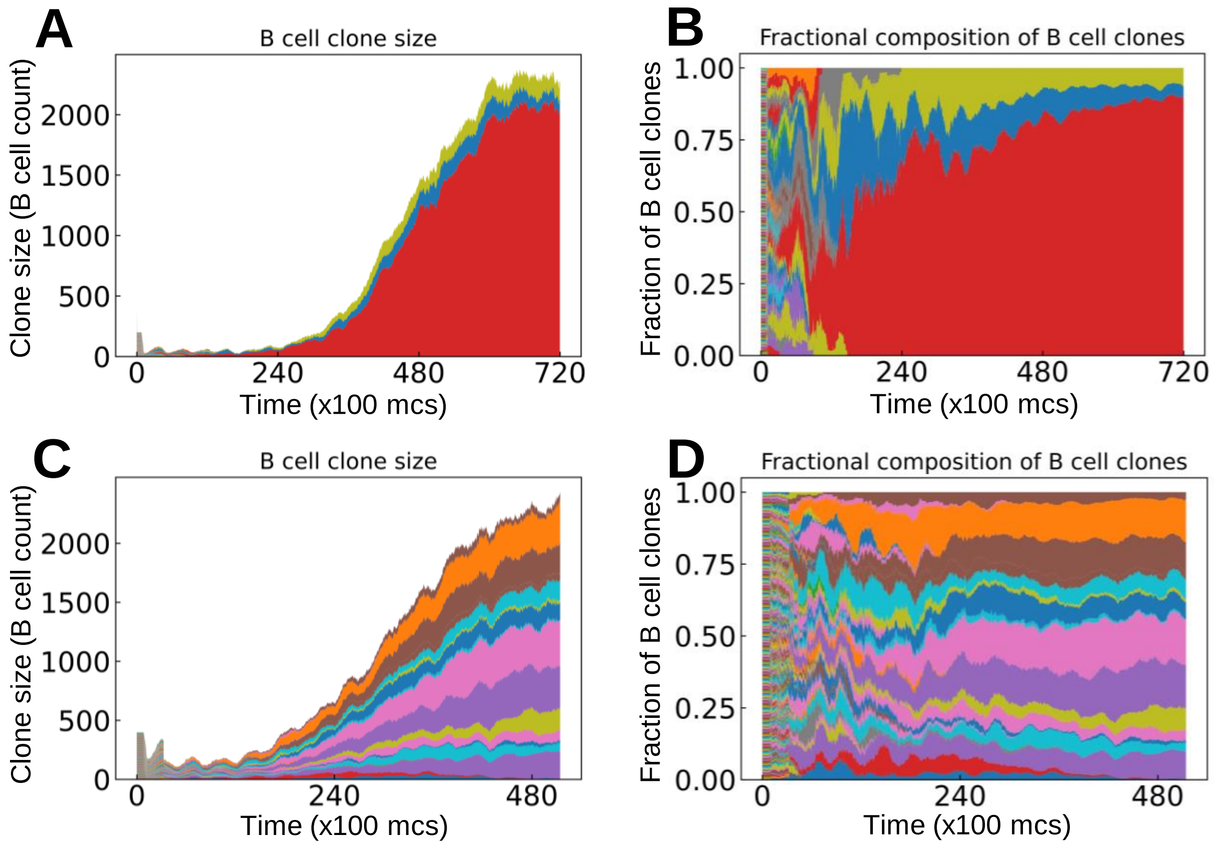

Figure S1. (A-B) Evolution of B cell clone sizes and clonal fractions respectively for a GC simulation resulting in single-clone dominance. **(C-D)** Evolution of B cell clone sizes and clonal fractions respectively for a GC simulation resulting in multi-clone dominance.

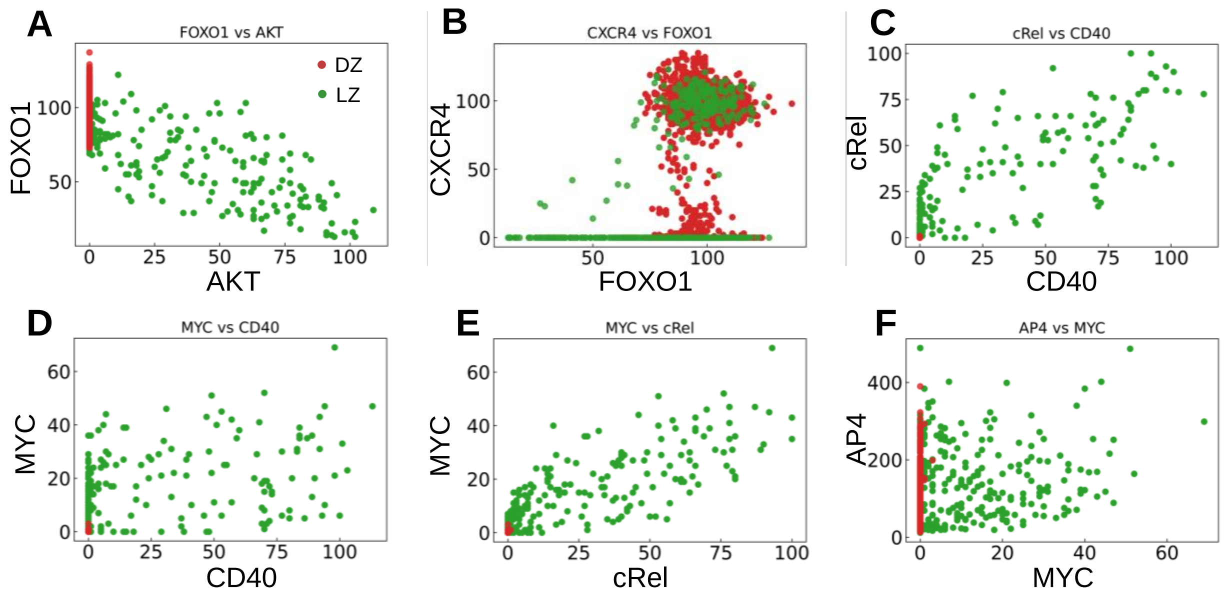

Figure S2. Correlations between key signaling molecules in DZ and LZ B cells as indicated at 70,000 *mcs*. Red and green dots denote DZ and LZ B cells respectively as shown in (A).

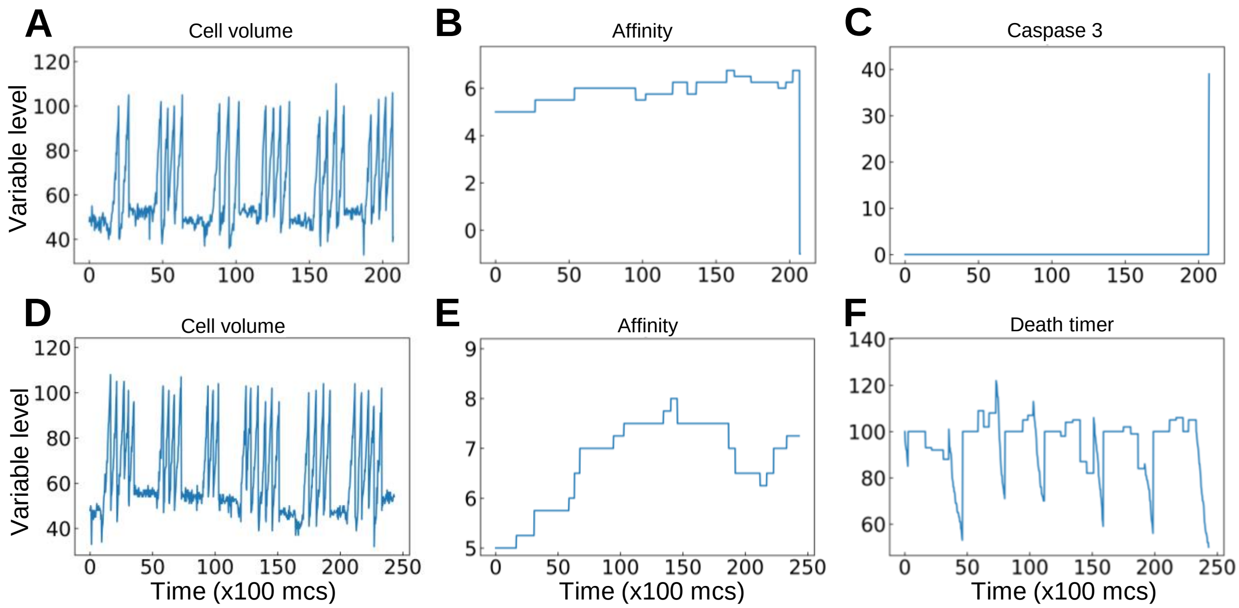

Figure S3. (A-C) Trajectory of a single B cell lineage branch that ends up in death in the DZ due to damaging BCR mutation. **(D-F)** Trajectory of a single B cell lineage branch that ends up in death in the LZ due to not being positively selected and the death timer counts down below a threshold level. Molecular and cellular variables are indicated.

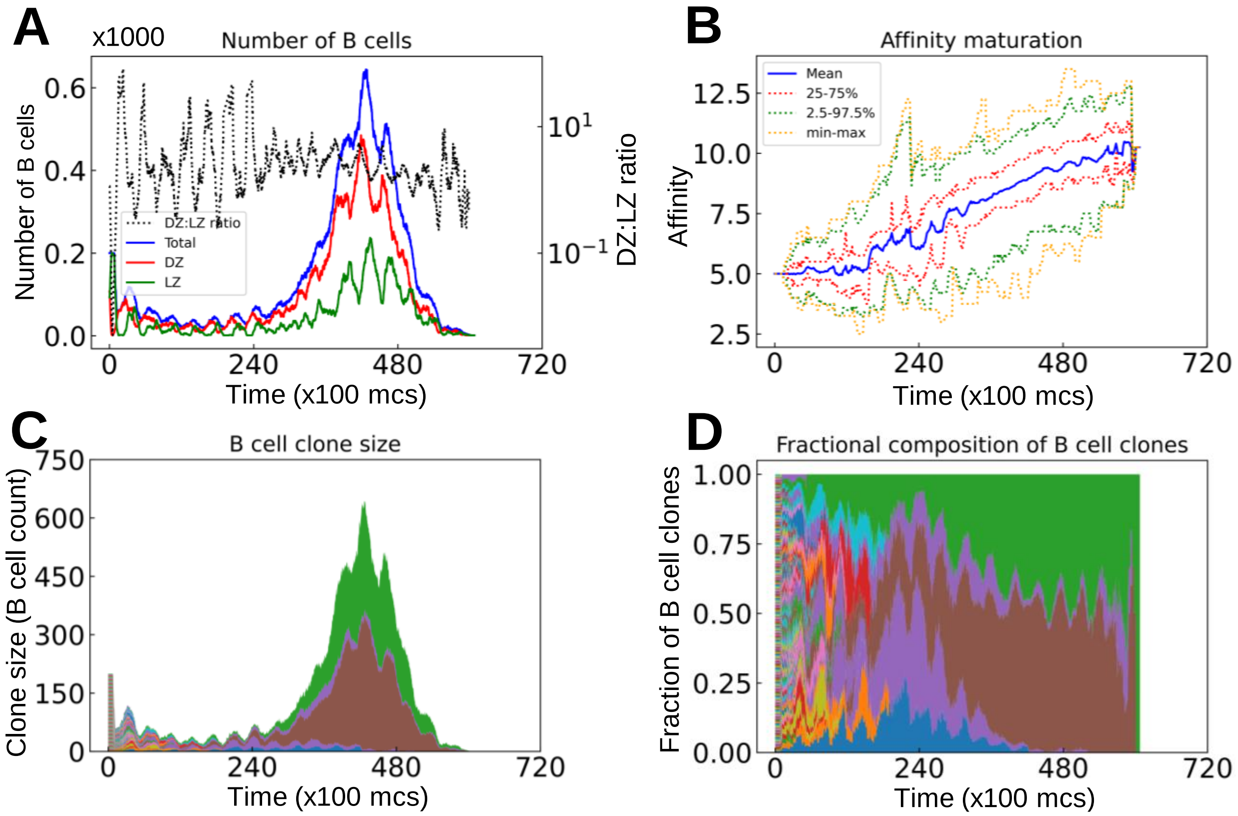

Figure S4. A simulated GC with self-termination. (A) Numbers of B cells in the DZ, LZ, and total, and DZ:LZ ratio as indicated at a given time. **(B)** Mean, interquartile, 2.5-97.5th percentile, minimum and maximum BCR affinities of GC B cells as indicated. **(C-D)** Evolution of B cell clone sizes and clonal fractions respectively. The GC termination is implemented via an FDC antigen depletion process – after 36,000 *mcs* the availability of antigen presented to B cells diminishes in a first-order (exponential) manner with a half-life of 10,000 *mcs*.

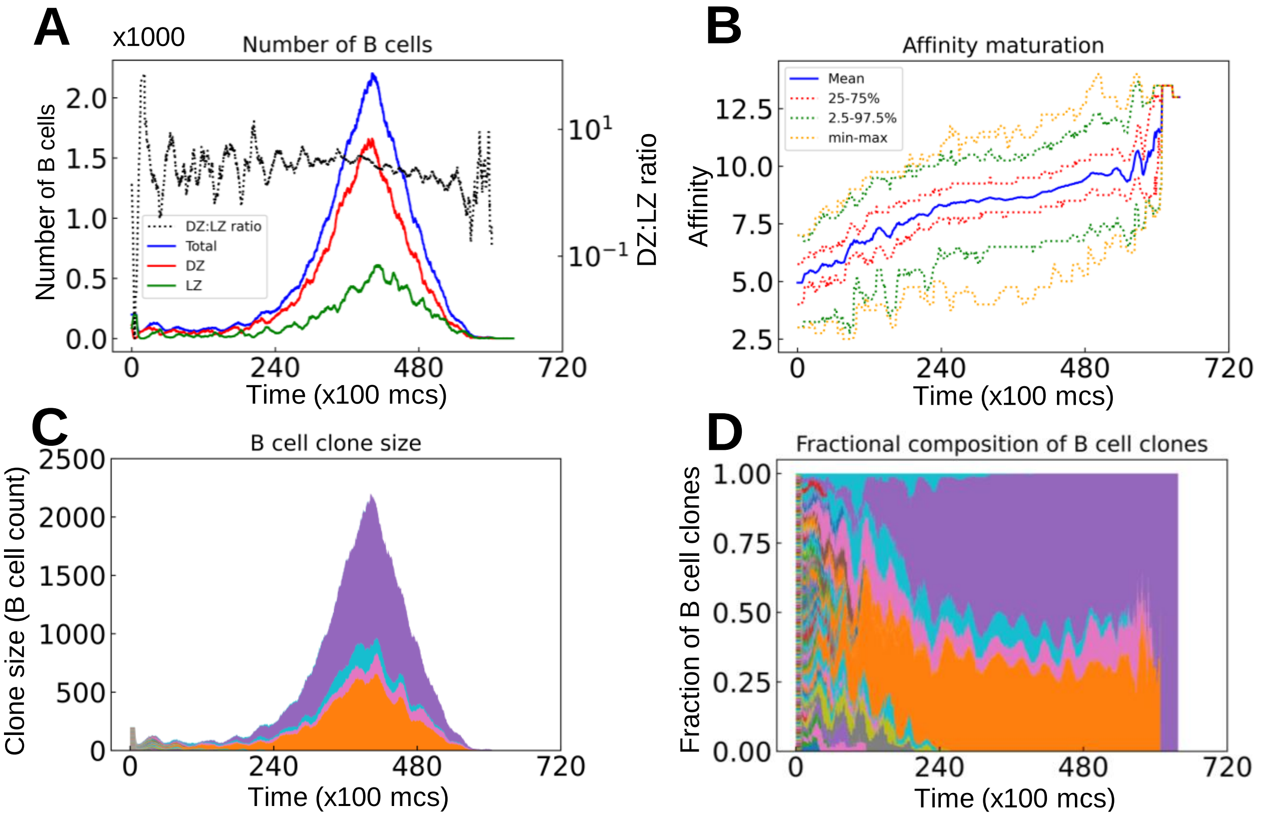

Figure S5. A simulated GC with varying initial affinity and self-termination. (A) Numbers of B cells in the DZ, LZ, and total, and DZ:LZ ratio as indicated at a given time. **(B)** Mean, interquartile, 2.5-97.5th percentile, minimum and maximum BCR affinities of GC B cells as indicated. **(C-D)** Evolution of B cell clone sizes and clonal fractions respectively. The initial affinities of the GC seeder B cells are varied by randomly drawing from a uniform distribution of [3-7]. The GC termination is implemented as in Fig. S4.
